# Microwave Monitoring of Atmospheric Corrosion of Interconnects

**DOI:** 10.1149/2.0181812jss

**Published:** 2018

**Authors:** Papa K. Amoah, Dmitry Veksler, Christopher E. Sunday, Stéphane Moreau, David Bouchu, Yaw S. Obeng

**Affiliations:** 1 Engineering Physics Division, Physical Measurement Laboratory, National Institute of Standards and Technology, Gaithersburg, Maryland 20899, USA; 2 CEA-LETI, MINATEC Campus, F-38054 Grenoble, France

## Abstract

Traditional metrology has been unable to adequately address the reliability needs of emerging integrated circuits at the nano scale; thus, new metrology and techniques are needed. In this paper, we use microwave propagation characteristics (insertion loss and dispersion) to study the atmospheric interconnect corrosion under accelerated stress conditions. The results presented in this work indicate that the corrosion resilience of the test device is limited by the thermal aging of the passivation layer.

The reliability challenges of emerging advanced integrated circuits nodes have not been adequately addressed at the nano-scale level by traditional metrology.^1,2^ For example, the stress buildup, due to thermal mismatch of the materials of construction, results in the generation of performance limiting defects,^3^ which are not readily detected with such techniques as DC-resistance changes. Physical analyses of devices that failed while in use suggest that damage in final encapsulation materials is a significant failure mode, probably due to the fact that the stresses in interconnects are influenced by the intrinsic properties of the passivation dielectrics.^4,5^ It has also been shown that the electromigration lifetime of interconnect metal lines is highly influenced by their proximity to the device encapsulating passivation layer.^6^ Furthermore, there is a persistent desire for alternate reliability techniques to help gain better mechanistic insights into device failure, and to reduce test time. Along these lines, highly accelerated stress tests (HAST) with high temperature and humidity with or without bias (e.g., JEDEC standard tests JESD22-A110, JESD22-A102, JESD22-A118, etc.) have been used.^7^ However, the metrology for monitoring these tests tend to be very complicated, and yield unsatisfactory reliability models^8^ which do not afford much mechanistic insight.^9,10^ Illustratively, it is incomplete to discuss the reliability of dielectric-cladded metal interconnect systems without considering the contribution of the metal-dielectric interface formed because of the adhesion of the dielectrics to the metal.^5,11,12^ The traditional metrology, however, does not directly provide this information. Thus, there is a need for better understanding of the physics of failure of final encapsulation materials and their impact on interconnect reliability. In general, new metrology techniques are needed to characterize failure modes, especially at the nano scale, and to relate them to the reliability of structure and composition.^13,14^

Heat induces changes in the electrical and physical properties of an interconnect, such as resistivity change due to crystallographic structural changes,^15^ oxidation, and void formation. Such alterations result in changes in the device’s impedance that are easily monitored with the insertion losses extracted from scattering parameters (S_12_ or S_21_) of broadband microwave spectrum. Furthermore, the phase changes in the propagating microwave signal can yield additional mechanistic information, such as film formation and chemical changes in dielectric properties of the materials of construction.^16^ Thus, it would be interesting to relate the chemical changes to mechanical artifacts such as void formation, decohesion, delamination, dielectric degradation, etc. of a material system (conductor, cladding, dielectric, processing condition etc.), to environmental factors. Such a link would provide rapid feedback for process and material integration optimization during device fabrication.^17^ Broadband microwave spectroscopy provides unique opportunities for probing and understanding the impact of thermal energy on devices. Heat induces vibration and eases the reorientation of polar moieties in changing electric fields. Such transitions show up in the insertion loss (S21) spectra and can be modeled with lumped-RLGC equivalent circuits.^18,19^ For integrated circuits on silicon substrates, simulations indicate that while isolation dielectric issues dominate low frequency (10 to 300 MHz) behavior, the resistance of the silicon substrate dominate 300 MHz-1 GHz region, and the capacitance of the silicon dominates the high frequency (> 1 GHz) region of the microwave spectrum as observed by increased insertion loss behavior, respectively.^18^ We have demonstrated that broadband microwave-based techniques afford detailed insights into the thermomechanical reliability issues,^20,16,21,22^ as well as to probe the impact of temperature and current on the pre-failure, in the early stages of EM of Cu through silicon via (TSV)-based interconnects in 3D-ICs.^23^ Both electromigration (EM) and corrosion failure modes manifest in increased interconnect direct current resistance (R_DC_); so, techniques are needed to distinguish between them.^20,24^ In this paper we characterize heat-induced atmospheric corrosion of metal interconnects, due to the failure of the encapsulating material, and attempt to distinguish it from other pre-electromigration (pre-EM) processes that occur during the latent period before catastrophic EM failure as described by Gousseau et al.^25^

## Experimental

### Materials.—

Dedicated ground-signal-ground (GSG) test structures were used in these experiments. The TSV-enabled devices under test (DUT) comprised of two-level stacked dies, with a Cu damascene redistribution level (RDL) similar to those described by Frank et al.,^26^ but encapsulated with a photo-definable thermoset polymer.^27^ The configuration of the test vehicle enabled a direct access to intrinsic reliability of TSV interconnects. In this test structure, the barrier between the TSV-fill and the bottom metal could become a flux divergence point.^28^ The “as received” devices were stored in a dry N_2_-box for at about 18 months during the studies.

**Figure 1.**
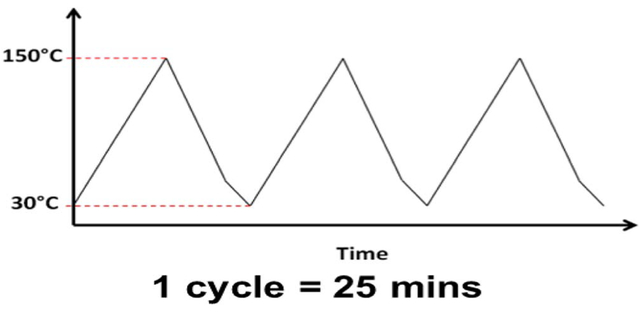
The thermal profile use in the dry thermal cycling test (TC-1).

**Figure 2.**
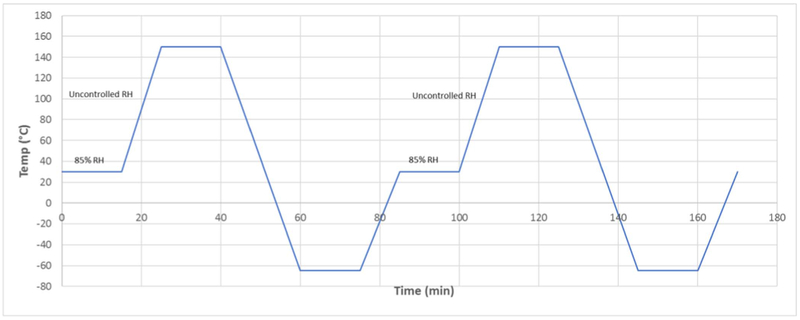
The thermal profile used in the modified-HAST test (TC-2). Each cycle is 76 minutes long.

In this paper, the thermo-mechanical reliability of the test samples was evaluated with two types of thermal cycling: a dry thermal cycling was conducted under dry N_2_ (dubbed TC-1), and a modified Highly-Accelerated Temperature and Humidity Stress Test (Unbiased HAST, JEDEC Standard JESD22-A118B, dubbed TC-2). The thermal profiles of the TC-1 and of the TC-2 are shown in Figures 1 and 2, respectively. These temperature profiles were chosen to mimic the environmental conditions that a typical chip will experience in service.

RF scattering data was obtained based on a two-port measurement on a vector network analyzer, using a setup in which the samples were placed on a heated chuck (managed to within ±1°C of target temperature) in an open laboratory ambient and direct current (DC) was forced through the signal line.^23^ Once the DUT reached the target temperature, and with the stress current still on, the microwave probe tips were landed on the DUT’s probe pads, and the RF signals continuously monitored with the 2-port network analyzer (PNA-L N5230C, 10 MHz- 40 GHz, Keysight, Inc., Santa Rosa, CA). A bias-tee was used to prevent the DC current from entering the vector network analyzer (VNA) circuit. The reference plane of the measurements was moved to the probe tips by following enhanced line-reflect-reflect-match (eLRRM) calibration using a well characterized impedance standard substrate (ISS) calibration substrate and WinCal XE algorithm (Cascade-Microtech, Beaverton, OR).^29,30^ A direct current measurement circuit was created in series with the DUT using a high-quality ceramic (1 Ω) resistor to simultaneously extract the DUT DC-resistance during the measurements. While we did not explicitly measure the expected additional Joule heating from the forced DC current through the DUT, we do not expect it to exceed 10°C, because of the limited number of interconnect levels and the current densities used in these studies, and as shown on similar test devices by Frank et al.^26,31,32^

## Results and Discussion

**Figure 3.**
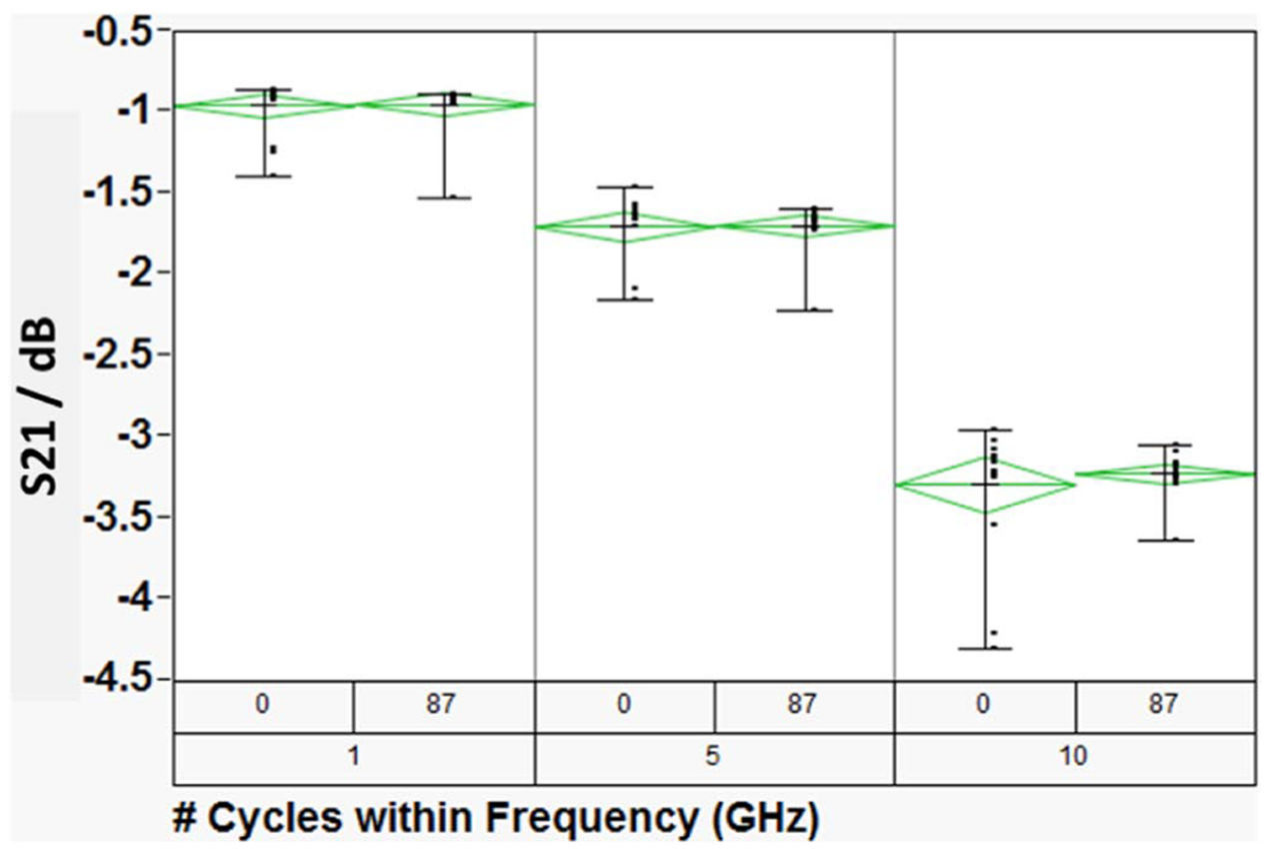
A comparison of the DUT’s insertion losses (S21) at three discrete frequencies ‘as-received’ and after 87 dry thermal (TC-1) cycles. The data bars represent the range of S21, while the green diamonds represent the statistical analysis of the data. The center line of the diamonds represents the mean, while the apex represents the one standard deviation of the data.

**Figure 4.**
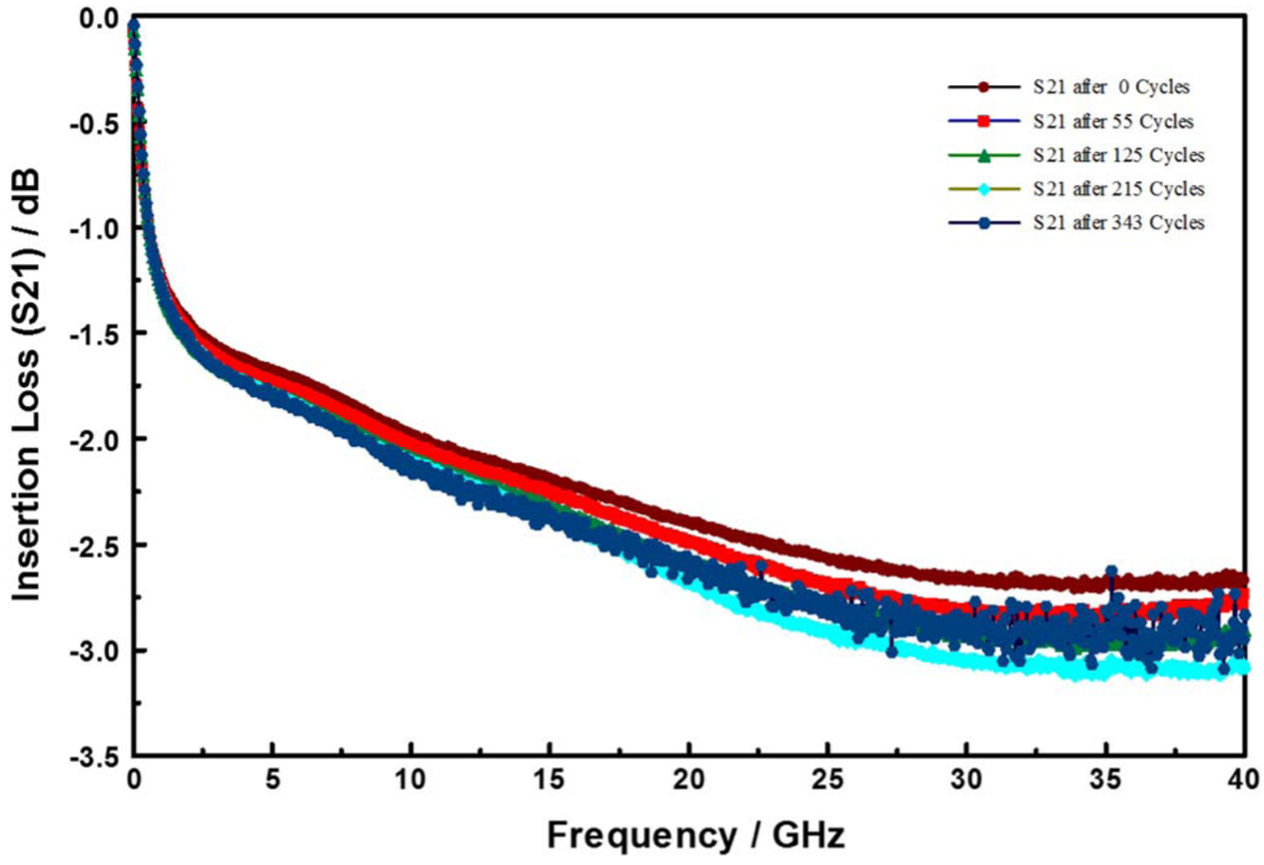
Evolution of insertion losses (S21) as a function of number of modified-HAST (TC-2) cycles.

**Figure 5.**
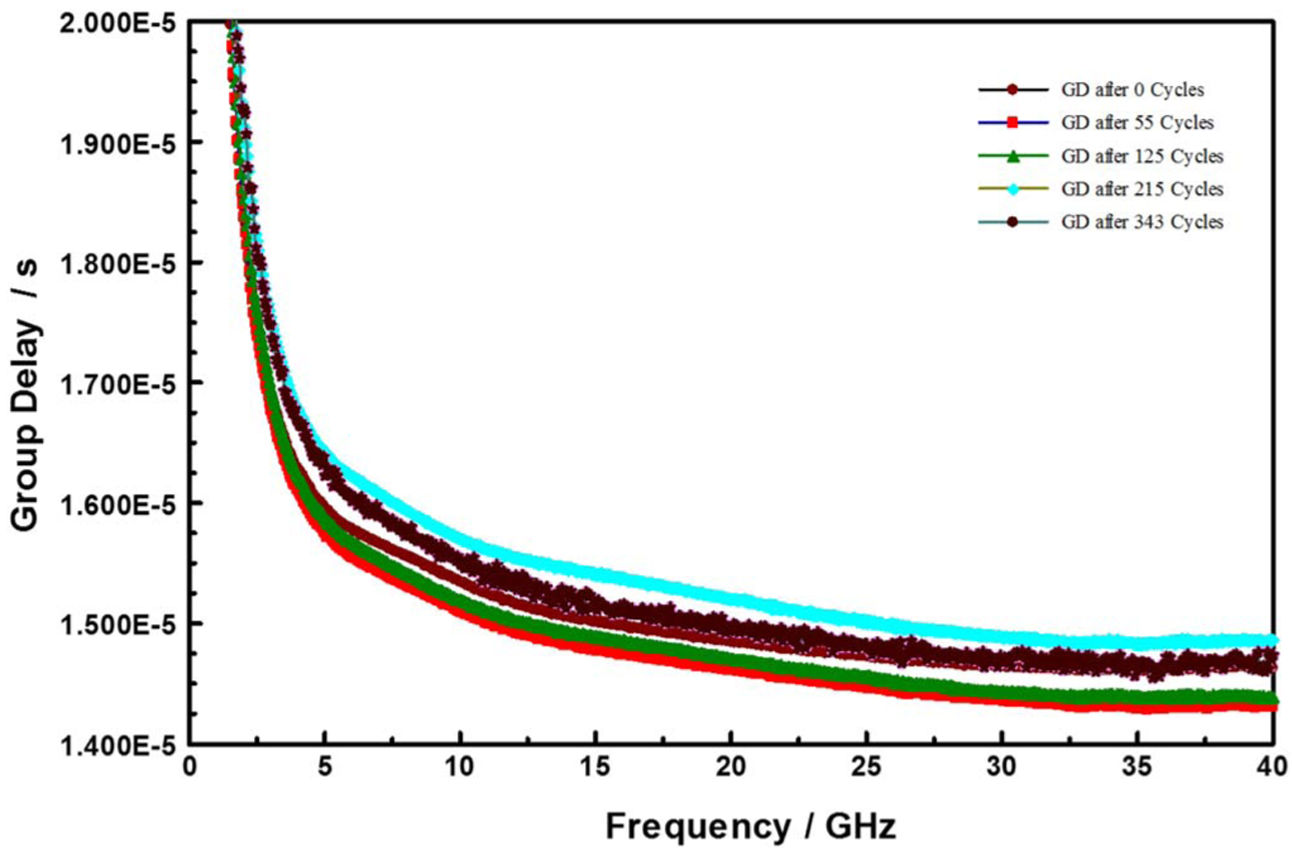
Dispersion in group delay (GD) as function number of modified-HAST (TC-2) cycles.

Insertion loss describes radiation losses from the materials of construction (i.e., metal, dielectric and their interfacial films), and is comprised of two parts: – dissipative (resistive) loss and reflection (mismatch) loss. In this work the dissipative loss (S21) appears to dominate the measurements. Thus, in the following we use S21 and insertion loss interchangeably to represent the dissipative loss. Figure 3 compares the insertion losses (S21) at three discrete frequencies, as a function of dry thermal cycling (TC-1). The TC-1 cycles were terminated because of catastrophic sample failure. In the limited experiments, the TC-1 did not result in any significant change in the electrical properties of the devices under test. On the other hand, as shown in Figure 4, the S21 decreased with increasing number of TC-2 cycles, and then became very noisy around 300 cycles. Furthermore, as shown in Figure 5, the microwave signal group delay, which effectively probes the details of the DUT’s dielectric response, also changed with increasing number of TC-2 thermal cycles. Notice, however, that the change in group delay was not monotonic, which suggests that materials of construction were changing, possibly through thermally driven chemical reactions, throughout the TC-2 cycling process.

**Figure 6.**
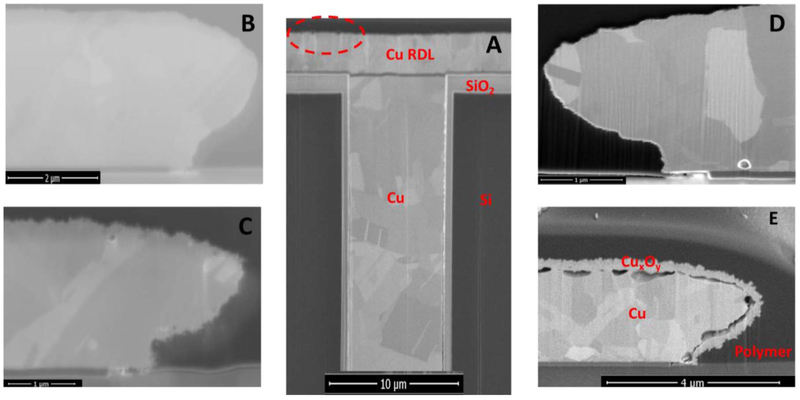
SEM micrographs showing the development of copper oxide films around interconnects as a function of stress temperature and time: (A) the dashed red circle is a visual aid showing the general location of magnified segment of interconnect, (B) “as-received” DUT, (C) after 343 TC-2 cycles (i.e., ~18 days), (D) 3 days at a static temperature of 125°C, and (E) after 4 days at a static temperature of 200°C. The thickness copper oxide film that formed around the Cu interconnect traces was measured from scanning electron micrographs (SEM) of focused ion beam (FIB) cross-sectioned samples.

The changes in the microwave loss spectra were most prominent at frequencies greater than 300 MHz, suggesting that the wet thermal cycling effects involve the metal and RDL materials, and not the TSVs. Optical inspection of the samples shows that the DUTs were visibly different after 200 cycles. The change in the insertion loss spectra, suggest significant mechanical damage or modification of the materials of construction with TC-2. This is supported by the extensive corrosion and mechanical damage to the RDL layer on the TC-2 samples as shown in Figure 6, which compares the cross-sectional electron micrographs of copper interconnect after a variety of thermal stresses. Such corrosion effects are most likely to be observed at mechanical defect sites in the encapsulation layers, with water adsorption or condensation on the surface of the interconnects.^33^

In addition, the encapsulating polymer materials can decompose or fail mechanically at high temperatures to expose the interconnect and influence the corrosion susceptibility of the device. In the absence of galvanic corrosion in the interconnect traces, the surface leakage current due to ionic corrosion products contamination, and hence a reasonable proxy for corrosion rate, is exponentially dependent upon ambient relative humidity above 40–50%.^34^ In microelectronic devices, electrolytic corrosion processes proceed at a rate that is determined by the electrochemical kinetics at the corroding metal. Thus, in unbiased devices, failure due to corrosion can be considered thermodynamically and modeled in terms of an Eyring type reaction rate for the multiple chemical reactions involved. However, in electrically biased samples, the rate-limiting step for interconnect degradation could increase with increasing current. Interestingly, increased currents also cause Joule heating which increases the local temperature and reduces the amount of adsorbed moisture, and the possibility of water ingress, at the corrosion site. Hence, under the test conditions used in these studies, there appears to be a competition between the temperature-induced encapsulant failure and the amount of corrosion-inducing adsorbed water.

**Figure 7.**
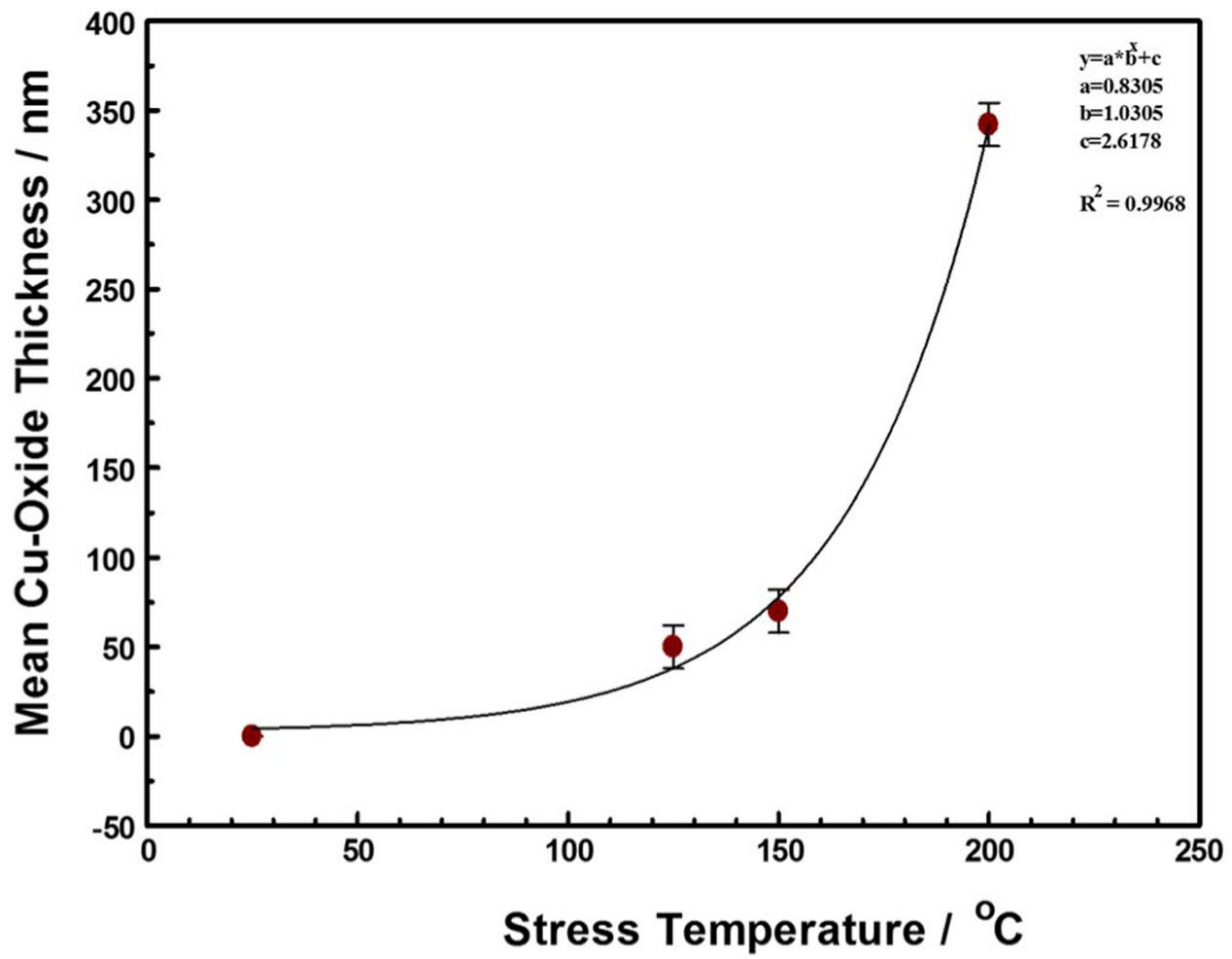
Thickness of copper oxide film formed around Cu interconnect trace as a function of stress temperature for at least 3 days. The copper-oxide film thickness was independent of stress time after 2 days. The error bars are entirely attributable to the different stress current used. The fit equation (insert) represents the relationship between the thickness of the copper oxide film around the copper RDL traces and the stress temperature.

The extent of oxidation, as measured by the thickness of the copper oxide lace formed around the copper trace, shown in Figure 6, increased with increasing temperature and duration of stress, as shown in Figure 7. Although the samples studied in this work were stressed for different durations, between 3 to 18 days, the measured copper oxide thickness did not show time dependence. Thus, the formation of the copper-oxide film appears to be self-limiting. This is consistent with Platzman et al. who divide the overall Cu-oxide thin film formation into three major stages: an initial formation of a copper (I) oxide layer, followed by a hydrolysis of the oxide-air interface to create metastable copper(II) hydroxide phase, and finally the transformation of the Cu(OH)_2_ metastable phase to the stable copper (II), CuO layer.^35^ The formation of the copper (I) oxide layer is induced by an electric field (as a driving force at low-temperature conditions) formed by positive ions of Cu at the metal/oxide interface and negative ions at the oxide/air interface. As the oxide layer thickness increases, the electric field drops across the film, and ceases to be strong enough to induce the metal cations to migrate, thus limiting the native copper-oxide film growth. The native copper oxide then undergoes chemical transformation toward the stable CuO film. Thus, although we did not chemically characterize the copper oxide films formed in this work, at temperatures below 300°C, we expect the copper oxidation product to be primarily Cu_2_O, with an outer coating of CuO.^36,37^

**Figure 8.**
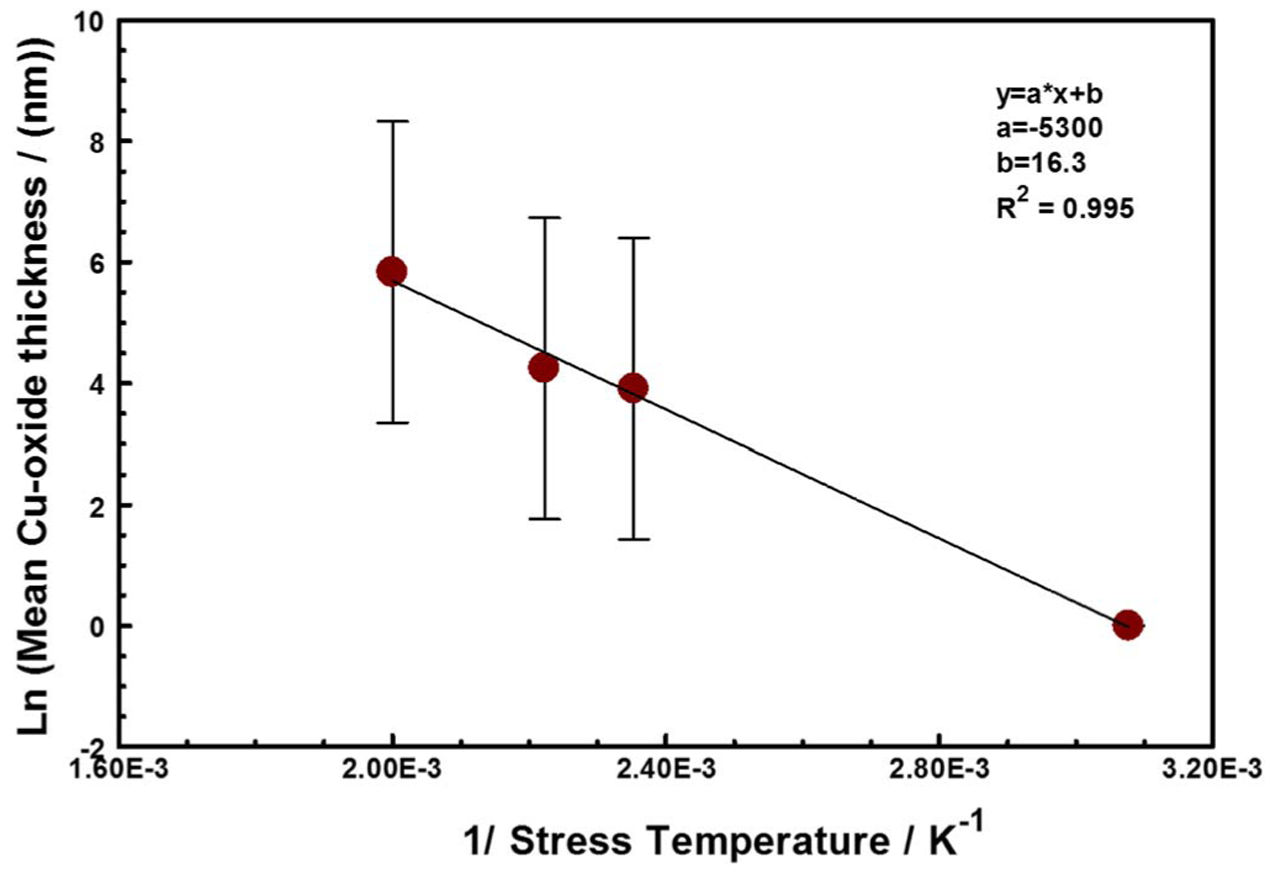
The Arrhenius fit of the copper oxide thickness (in nanometers) as a function of stress temperature. The activation energy for the copper oxide growth was calculated to be about 4.4 kJ/ mole with a correlation coefficient (R^2^) of 0.995.

The activation energy for the copper oxide growth, calculated from the Arrhenius fit in Figure 8 is about 0.05 eV (i.e., 4.4 kJ/mol). This value is too low for the expected activation energy for the atmospheric oxidation of copper at temperatures above 100°C, which is in the 1.60 eV to 1.75 eV range.^38^ The mechanism of atmospheric oxidation of copper appears to strongly temperature dependent.^39,40^ Above 75°C to 100°C, the oxidation process is thermally activated, with an activation energy in the range of 1.60–1.75 eV. Below the 75°C, the initial nucleation and growth of the copper oxide film appears to be athermal.^38^ The activation energy we report here is suggestive of spontaneous dissociative chemisorption of water molecules on the copper surface,^41^ following the rupture of the encapsulating thermoset polymer. Thus, in this study, the oxidation of the copper interconnects appears to be gated by the breach in polymer encapsulant which allowed water / corrosive species ingress and adsorption on the interconnect metal, and between the Cu RDL and the passivation interface. The breach increases with increasing stress temperatures, as seen in Figure 6. Once the encapsulant fails, the copper interconnects oxidize rapidly due to the adsorption of water from the ambient air.^42,36^

**Figure 9.**
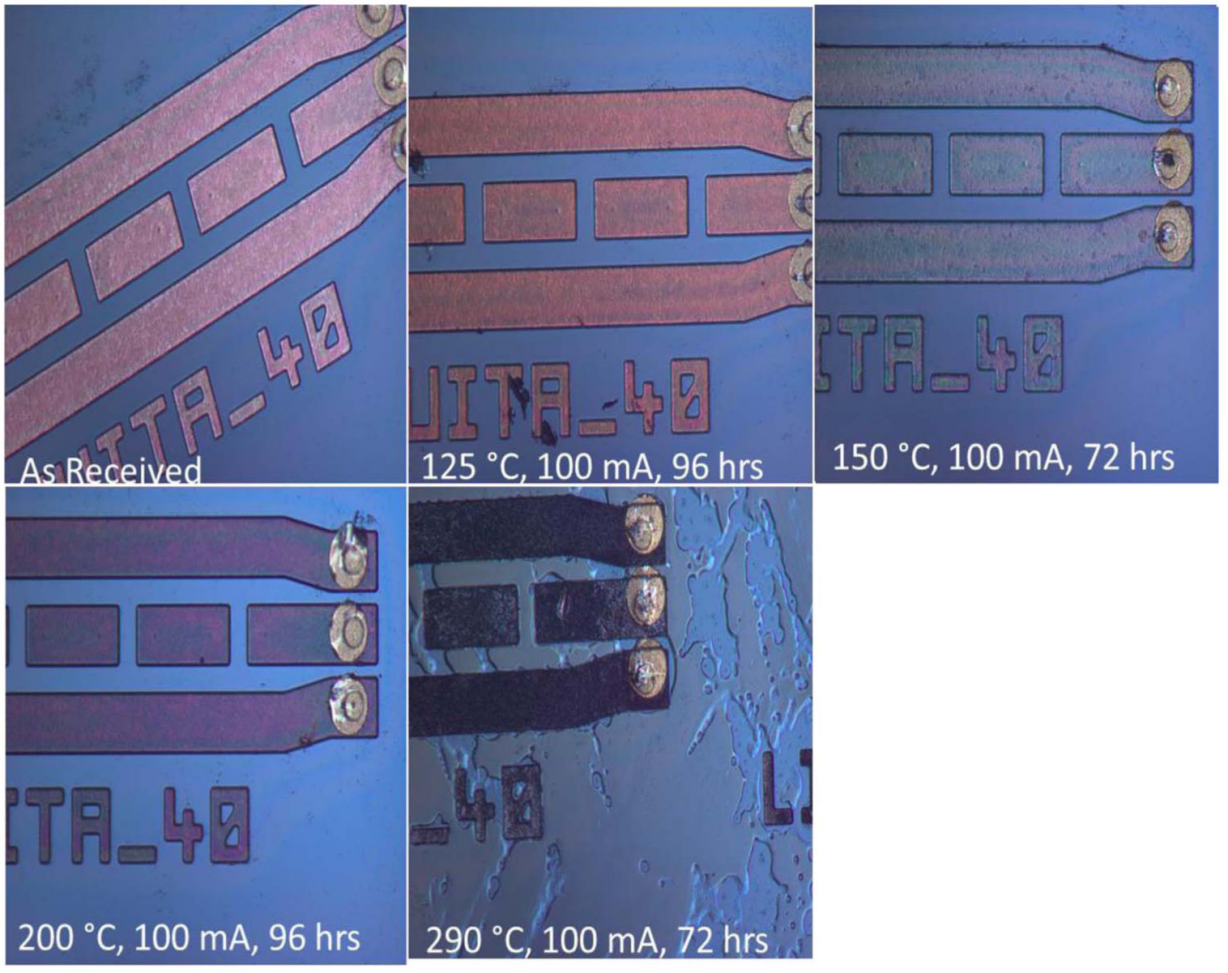
Room temperature optical micrographs showing color changes of the Cu RDL as the DUT is heated to different temperatures. Notice the decohesion in the polymer coating in sample heated to 290°C for 72 h.

Heating the DUT to temperatures greater than 250°C, resulted in color changes of the RDL. Figure 9 compares the room temperature optical micrographs of test devices heated to various temperatures for at least 3 days. Significant decohesion/puddling of the polymer coating was observed in the sample heated to 290°C for 72 h, which is consistent with the known passivation polymer’s glass transition temperature. The color change may be attributed to thermal oxidation of organic functional groups leading to chain scission and discoloration in the encapsulating polymer.^43^

**Figure 10.**
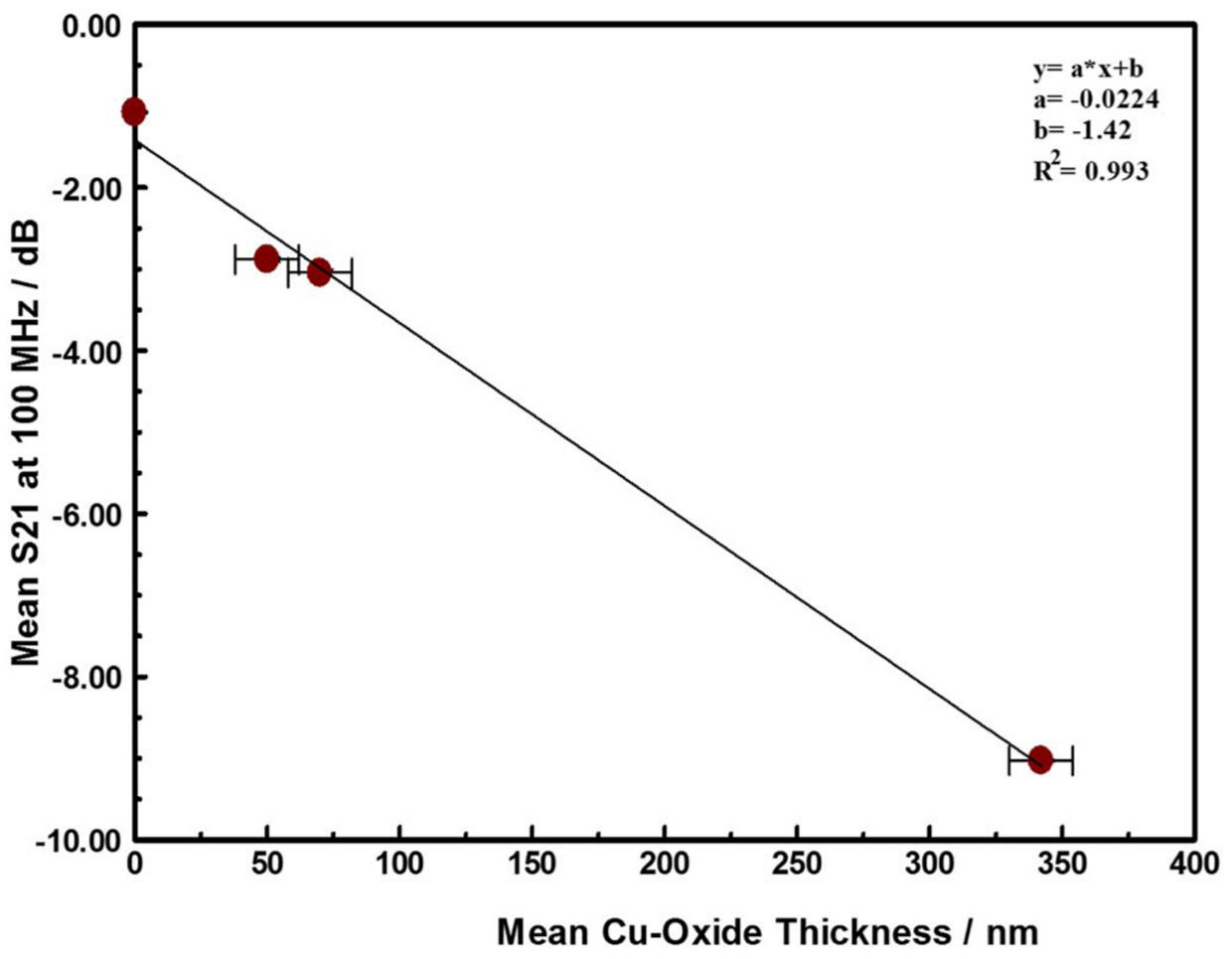
Correlation between the thickness of copper oxide film formed around Cu interconnect trace in samples maintained at stress temperature for at least 3 days and the Insertion loss (S21) at 100 MHz. The intercept on the y-axis represents the room temperature insertion loss of the as received material, prior to ant stress.

The copper oxide film formed from the oxidation of the copper was monitored by the microwave insertion loss (S21) at 100 MHz. Figure 10 compares the S21 at 100 MHz to the thickness of the copper-oxide film formed around the copper interconnects. Increasing the stress temperature resulted in increased transmission loss (S21) due to increased electrical resistance (R_DC_) as the increasing thickness of the copper-oxide film that form around the copper interconnects reduces the conduction path. In this paper, the measured copper oxide thickness is very weakly dependent on the magnitude of the forced current, and independent of the stress time.

**Figure 11.**
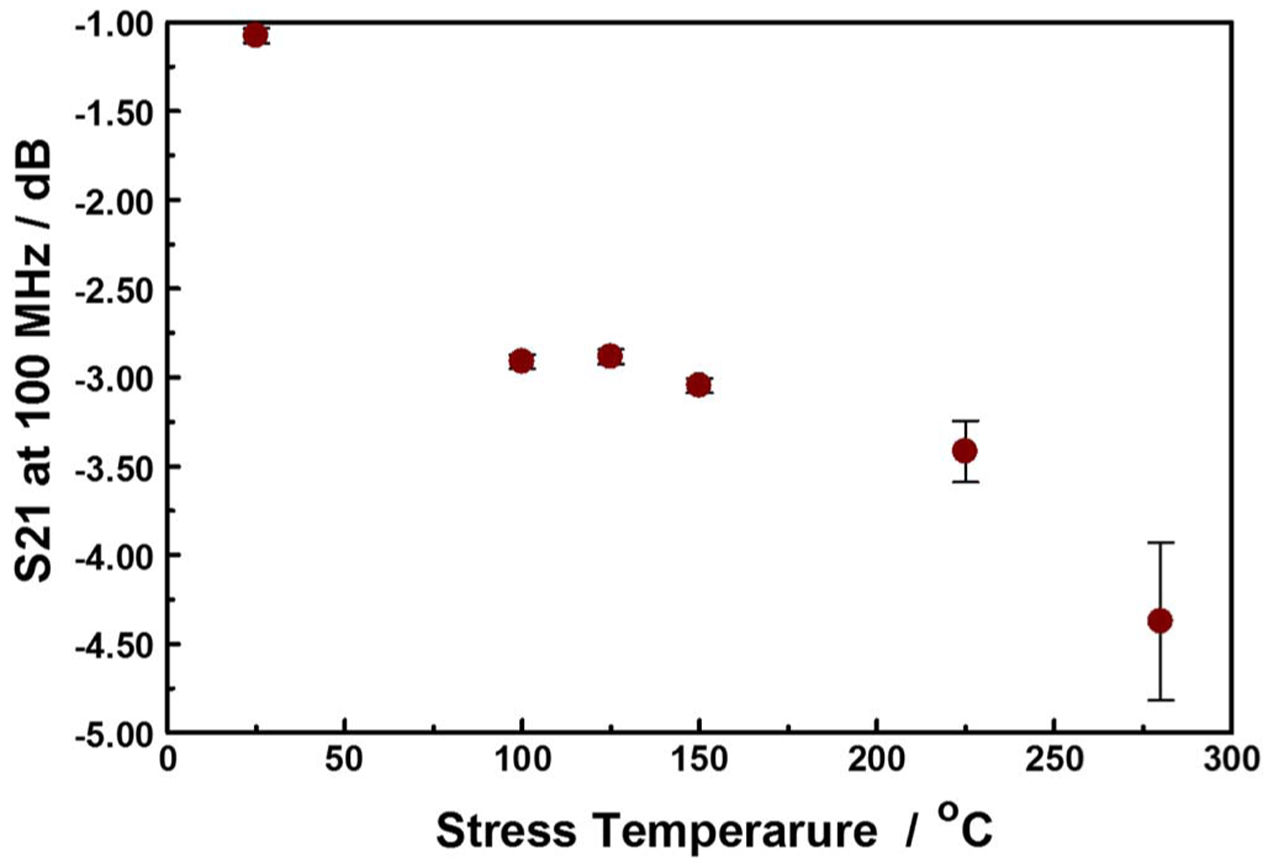
Evolution of the S21 at 100 MHz with device stress temperature for at least 3 days. The copper-oxide film thickness was independent of stress time after 2 days. The error bars are entirely attributable to the different stress current used.

Using the S21 as an index for copper oxide film thickness, we can monitor the extent of corrosion RDL as a function of stress temperature.Figure 11 shows the evolution of the S21 at 100MHz as a function of device stress temperature. The plot shows a sigmoidal trace, indicating that there are at least two, probably competing, processes in the range of temperature studied. This data complements our previous study in which the S21 appears to recover if the stress is maintained for a period shorter than 48 hours.^23,44^ In this work, the insertion losses did not recover if the thermal stress is maintained for at least 48 hours, irrespective of magnitude of the forced current. Such that, taken together the data suggest that the initial corrosion process is reversible (i.e., elastic), while the later ones are not (i.e., plastic). Temperature-driven transformations within the isolation dielectrics are less likely to be responsible for the elastic processes as they are irreversible.^16^ However, when considered in view of the low activation energies the reversibility of the initial stages is probably related to reversible processes within the encapsulating polymer layer, and/or formation of metastable oxidation products. On the other hand, the plastic losses are attributable to temperature driven degradation of the encapsulating polymer, and the spontaneous formation of stable copper oxide films on the the exposed copper lines, leading to permanent transformation in the DUT’s conduction pathways. The elastic to plastic S21 loss behavior suggest that the interconnect failure mechanisms are different for the short- (< 48 hours) and long-time (> 48 hours) domains. In the long-time (> 48 hours) stressed devices, the microwave insertion loss mechanisms are the result of gating thermally driven events., such as encapsulating polymer degradation.

**Figure 12.**
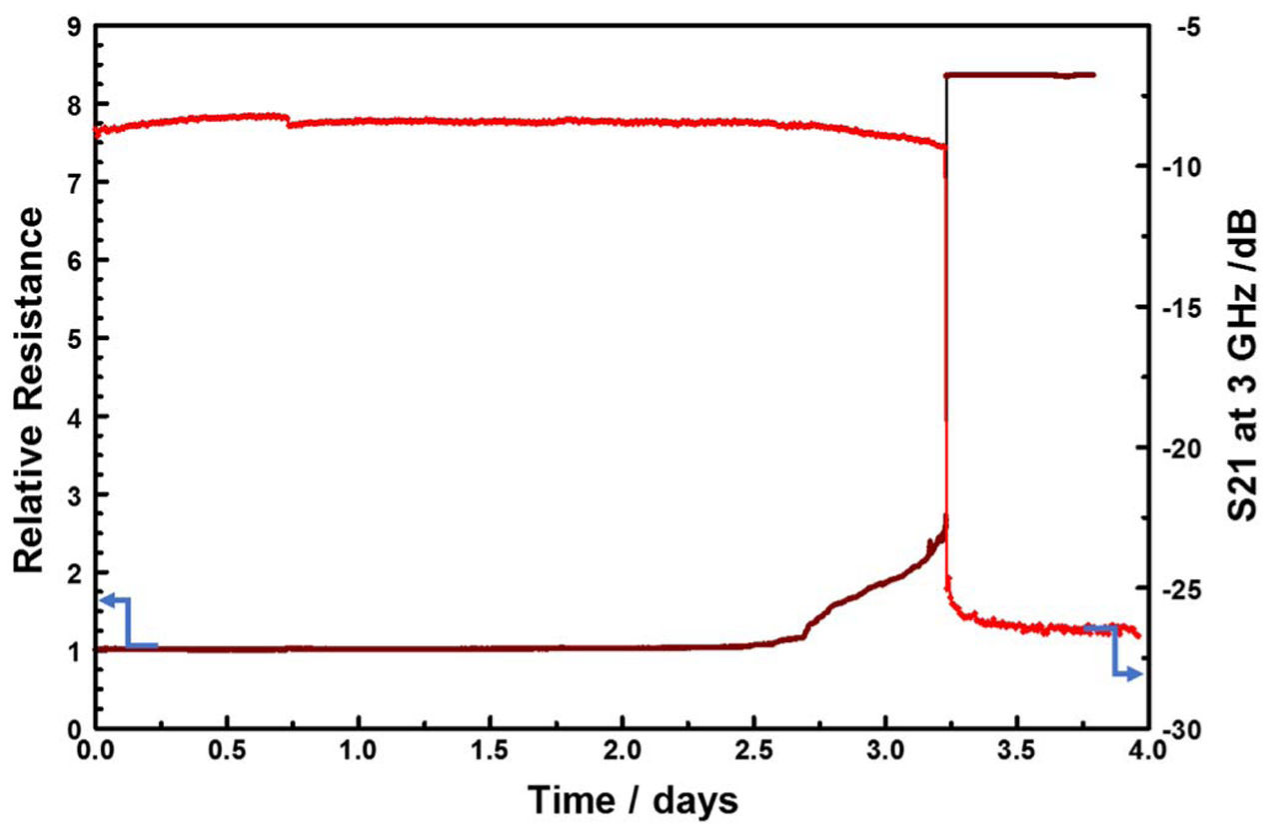
Comparison of the time evolution of relative-resistance and S21 insertion loss (at 3 GHz) over a 4-day EM stress test (forcing 100 mA and 290°C).

The idea of permanent change in the interconnect resistance after long stress times is further supported by the evolution of the measured direct current resistance (R_DC_) over time. As shown in Figure 12, there is good correlation between the time evolution of insertion losses(S21) and that of R_DC_. The resistance increase is attributable to the corrosion of the interconnect which reduces the current conduction path cross-section, as the copper is oxidized, forcing the current to go through the highly resistive TiN barrier which acts as a shunt layer. Close inspection of the R_DC_ trace in Figure 12 shows a small step increase in the resistance, starting at the 2.75-day mark, before the steep rise in resistance begins at the 3.25-day mark. This may be indicative of a transformation that precedes the corrosion of the metals line, such as passivation layer breach allowing water adsorption on the metal surface.^45,39^

**Figure 13.**
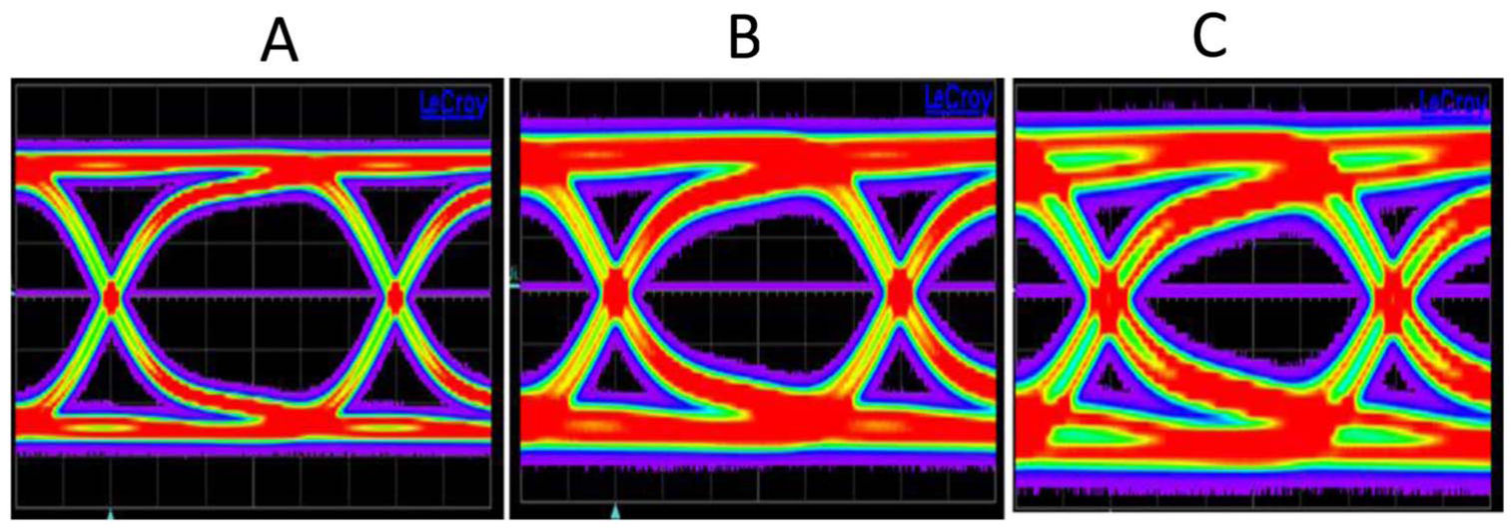
Comparison of PRBS15 pattern (500mV amplitude, 100 ps unit interval) eye diagrams of (A) calibration substrate, (B) “as received” and (C) highly resistive DUT after extensive stress test (6 days under 100 mA at 290°C stress).

In the following, we discuss the signal integrity consequences of the RDL corrosion on the capacitance-dominated TSV enabled interconnects structures.^46^ The Figure 13 compares the measured PRBS15 pattern eye diagram of (A) calibration substrate, (B) “as received” to (C) highly resistive DUT after EM test (6 days under 100 mA at 290°C stress). In the unstressed test devices, time domain 10-Gbps pseudorandom bit sequence (PRBS) can be propagated without significant distortion. However, the signal propagation in the thermally stressed devices suffers significant distortion due to changes in impedance matching, attributable to increased capacitance loading.^19^ The larger capacitance requires longer RC charging times, which results in eye opening deterioration.^47^ Such results are expected from the group delay changes observed with thermal stressing, such as summarized in Figure 5.

The changes in the eye diagrams are indicative of DUT mismatch to the reference impedance of the measurement bridge. Ordinally, such mismatch would present as increases in the return loss (S11); thus, we had expected increasing S11 with increasing copper oxide thickness. However, we did not observe such systematic increase in S11 with stress time or temperature, indicating that the changes in signal integrity may be the result of a complicated mechanistic regime, including human handling issues.^48^

## Conclusions

Changes in microwave signal scattering occur when copper interconnects corrode, principally due to device resistance increase from oxidation of metal lines. Pre-existing mechanical damage such as thermally driven passivation failure, appear to gate the metal oxidation rate. The insertion losses (S21) appears to correlate with the thickness of the of the metal oxide corrosion. Thus, the measurements reported in this paper allow us to directly observe resistive changes in insertion loss due to materials transformation, e.g., formation of resistive metal oxides due to corrosion. The potential applications of the insertion loss method include rapid detection of corrosion, evaluation of encapsulation materials and passivation processes, and prediction of interconnect lifetime under in harsh environments. We may also be able use the microwave signal propagation characteristics to distinguish between corrosion and electromigration failure modes.

It must be emphasized that whereas we have attributed the observed insertion loss predominantly to dissipative loss, the total loss mechanism is not purely resistive. There are many mechanisms that contribute to the observed radiation loss, and further work is needed to resolve the contributing mechanisms.
